# Efficacy of acupuncture combined with exercise rehabilitation on cardiac function in patients with heart failure: a systematic review and meta-analysis

**DOI:** 10.3389/fmed.2026.1789189

**Published:** 2026-07-02

**Authors:** Jiale Hu, Hongsen Du, Ting Chen, Yuanhui Hu, Na Huang, Zhen Ma

**Affiliations:** 1 Department of Cardiology, Xi'an Hospital of Traditional Chinese Medicine, Xi'an, China; 2 The First Clinical Medical College, Shaanxi University of Traditional Chinese Medicine, Xianyang, China; 3 Department of Cardiology, Guang'anmen Hospital, China Academy of Chinese Medical Sciences, Beijing, China

**Keywords:** acupuncture, cardiac function, exercise, heart failure, meta-analysis, randomized controlled trials

## Abstract

**Background:**

Exercise rehabilitation (ER) is recommended in both national and international guidelines for heart failure (HF). However, patients with severe symptoms may derive limited benefit from ER alone. Acupuncture has shown potential cardioprotective and symptom-relieving effects in some clinical studies, suggesting that acupuncture combined with exercise rehabilitation (ACER) may be a promising adjunctive strategy. This systematic review aimed to compare the efficacy of ACER with that of ER alone in patients with HF.

**Methods:**

The Cochrane Library, PubMed, Ovid, Wanfang Medical Database, China Biomedical Literature Service (SinoMed), Weipu Database (VIP), China National Knowledge Infrastructure (CNKI), http://ClinicalTrials.gov, and the International Clinical Trials Registry Platform (ICTRP) were searched from inception to March 2026. This review followed the PRISMA statement and assessed evidence with GRADE. The aim was to identify randomized controlled trials (RCTs) investigating the effects of ACER on cardiac function in patients with HF. Two investigators independently screened studies, extracted relevant data, and assessed the methodological quality using the Risk of Bias version 2 tool (RoB 2). Meta-analyses were performed using RevMan 5.3. The protocol was registered in PROSPERO (CRD420251184719).

**Results:**

Eight RCTs involving 668 patients were included, seven conducted in China and one in Serbia. Compared with ER alone, ACER was associated with statistically significant improvements in left ventricular ejection fraction [LVEFl; *MD* = 4.98, 95% CI (2.77, 7.18)], 6-min walk distance [6MWD; *MD* = 77.78 m, 95% CI (59.23, 96.32)], left ventricular end-systolic diameter (LVESD) [*MD* = −5.27 mm, 95% CI (−7.83, −2.71)], Minnesota Living with Heart Failure Questionnaire (MLHFQ) score [*MD* = −6.41, 95% CI (−7.71, −5.10)], and left ventricular end-diastolic diameter (LVEDD) [*MD* = −5.65 mm, 95% CI (−6.81, −4.50)]. However, the certainty of the evidence was low or very low across all outcomes because of high risk of bias, substantial unexplained heterogeneity, imprecision, and uncertain publication bias. These findings should therefore be interpreted with extreme caution.

**Conclusion:**

The current evidence is insufficient to determine whether ACER provides clinically meaningful benefits over ER alone in patients with HF. Further high-quality RCTs are needed before ACER can be recommended as an evidence-based adjunct in HF rehabilitation.

**Systematic review registration:**

https://www.crd.york.ac.uk/PROSPERO/view/CRD420251184719, identifier: CRD420251184719.

## 1 Introduction

Heart failure (HF) represents the end stage of various cardiovascular diseases and is associated with high mortality and poor quality of life, posing a major global public health challenge (1). Epidemiological data indicate that in 2017, HF prevalence was highest in Central Europe, North Africa, and the Middle East (1,133–1,196 per 100,000), whereas East and Southeast Asia had lower rates (498–595 per 100,000). HF is the leading cause of hospitalization among adults aged 65 years and older, accounting for 1%−2% of all hospitalizations in the Western countries (2). Between 2017 and 2021, the 1-year all-cause mortality rate after discharge was 13.7% among patients with HF in China (3). In the United States, HF-related mortality has risen steadily since 2012. By 2022, HF contributed to 425,000 deaths, representing approximately 45% of all cardiovascular deaths. Direct medical costs attributable to HF in the United States are projected to reach 142 billion by 2050 (4). Collectively, HF substantially impairs quality of life and places an increasing burden on healthcare systems (5), necessitating safe and effective therapeutic strategies.

Exercise rehabilitation (ER) is a cornerstone of HF management (6), improving peak oxygen uptake and quality of life while reducing hospital readmissions and mortality (7–10). However, its clinical implementation is often limited by poor tolerance and inadequate adherence (11). Acupuncture may alleviate cardiac symptoms and is considered a promising adjunctive therapy (12), potentially providing synergistic benefits when combined with exercise. Modern research suggest that acupuncture may supports cardiac structure and function by regulating vascular endothelium, oxidative stress, inflammation, apoptosis, and autophagy (13). A sham-controlled pilot trial also reported improved exercise tolerance, ventilatory efficiency, and post-exercise recovery with adjunctive acupuncture in patients with HF (14).

Although several studies have evaluated this combined therapy in patients with HF, no meta-analysis has synthesized the relevant evidence. This study therefore aimed to address that gap and provide an reference for the integration of traditional Chinese and Western medicine in HF management.

## 2 Materials and methods

### 2.1 Protocol and registration

This systematic review and meta-analysis followed the Preferred Reporting Items for Systematic Reviews and Meta-Analyses (PRISMA) statement, and the PRISMA 2020 checklist is provided in Supplementary Table S1. The study was prospectively registered in PROSPERO (CRD420251184719; registered on November 21, 2025). The review adhered strictly to the pre-specified protocol with no deviations. The full protocol is available at https://www.crd.york.ac.uk/PROSPERO/view/CRD420251184719.

### 2.2 Search strategy

A comprehensive search was performed across multiple databases, including the Cochrane Library, PubMed, Ovid, Wanfang Medical Database, China Biomedical Literature Service (SinoMed), Weipu Database (VIP), China National Knowledge Infrastructure (CNKI), http://ClinicalTrials.gov, and the International Clinical Trials Registry Platform (ICTRP) from the inception to March 2026. The search terms included “heart failure,” “cardiac failure,” “acupuncture,” “exercise,” and “randomized controlled trial.” The full search strategy is available in Supplementary Table S2.

### 2.3 Eligibility criteria

Inclusion criteria include (1) Study type: RCTs evaluating acupuncture combined with exercise rehabilitation (ACER) for HF, with no language restrictions. (2) Population: patients with a confirmed diagnosis of HF, including heart failure with preserved ejection fraction (HFpEF) or reduced ejection fraction (HFrEF), diagnosed according to the 2023 ESC Guidelines for the Diagnosis and Treatment of Acute and Chronic Heart Failure (15); no restrictions on age, gender, or nationality. (3) Interventions: the intervention group received ACER. For this review, ACER is defined as any invasive acupuncture modality (e.g., manual acupuncture, electroacupuncture, warming needle moxibustion, ear acupuncture, wrist-ankle acupuncture, or fire acupuncture) combined with any form of exercise-based rehabilitation (e.g., resistance training, flexibility exercise, aerobic exercise, or traditional Chinese exercises). Traditional Chinese exercises are mind-body practices rooted in traditional Chinese medicine (TCM) theory, including Tai Chi, Baduanjin, and Wuqinxi. (4) Comparators: the control group received ER alone (as defined above). (5) Outcomes: left ventricular ejection fraction (LVEF), 6-min walk distance (6MWD), Minnesota Living with Heart Failure Questionnaire (MLHFQ) score, left ventricular end-systolic dimension (LVESD), and left ventricular end-diastolic dimension (LVEDD).

Exclusion criteria include (1) The intervention group received acupuncture combined with any other therapy beyond ER, such as oral herbal formulas, proprietary Chinese medicines, cupping, or other complementary modalities. (2) Acupuncture used as a sole intervention. (3) Non-RCTs, animal studies, systematic reviews, meta-analyses, dissertations, mechanistic studies, or conference abstracts. (4) Studies with incomplete outcome data or unavailable full texts.

### 2.4 Data extraction and quality assessment

NoteExpress was used to compile, remove duplicates, screen the literature, and extract research data. Two independent reviewers conducted an initial screening based on titles and abstracts, followed by full-text assessment. Agreement between reviewers was evaluated using Cohen's kappa statistic. Any disagreements were submitted to a designated corresponding author for arbitration. Finally, data such as country of study, author names, publication years, funding sources for the studies, sample sizes, interventions details, New York Heart Association (NYHA) functional classification, treatment duration, and outcome indicators were extracted.

The risk of bias for the included RCTs was independently assessed by two reviewers using the Risk of Bias version 2 tool (RoB 2) (16) for the following domains: randomization process, deviations from intended interventions, missing outcome data, measurement of the outcome, and selection of the reported result. Each domain was judged as “low risk,” “some concerns,” or “high risk.” Disagreements were resolved through discussion or consultation with a third reviewer. The quality of evidence was assessed using the GRADE framework, and the following areas were included: indirectness, imprecision, risk of bias, inconsistency, publication bias, and other considerations. The quality of evidence was categorized as “high”, “moderate”, “low”, or “very low” quality. Specific evaluation rules are listed in the Supplementary Appendix S1.

### 2.5 Statistical analysis

RevMan 5.3 software was used for statistical analysis. Continuous variables were presented as mean difference (*MD*) with 95% confidence interval (CI). Dichotomous variables were statistically analyzed with odds ratio (OR) and 95% CI.

#### 2.5.1 Handling of missing data

For incomplete outcome data, we contacted authors. If unsuccessful, we assessed the impact of missing data. For studies reporting outcomes as medians and interquartile range (IQR), means and standard deviation (SD) were estimated using the method described by Wan et al. (17). Sensitivity analyses excluding studies with imputed data were conducted to assess the robustness of the pooled estimates. The risk of bias related to missing data was assessed for each study using RoB 2.

#### 2.5.2 Assessment of heterogeneity

Heterogeneity was assessed using the chi-square test with α = 0.1, and the degree of heterogeneity was evaluated based on the *I*^2^ value. This study reported *I*^2^ using the following general rules: 0 to 25% indicates low heterogeneity, 26 to 50% moderate, and 51 to 75% substantial heterogeneity. While we report *I*^2^ values, we acknowledge that *I*^2^ can be high even if the magnitude of inconsistency is not clinically important, particularly when study estimates are precise, such as when confidence intervals are narrow. Therefore, we also considered visual inspection of forest plots and the direction and magnitude of effect estimates across studies (18).

#### 2.5.3 Model selection

The choice between fixed-effects and random-effects models was guided by both statistical and clinical considerations. Given the anticipated variability in acupuncture protocols, exercise regimens, and patient characteristics, a random effects model was pre-specified as the primary approach. It was used if the chi-square test was significant (*P* < 0.1) and *I*^2^ > 50%, and heterogeneity could not be explained by subgroup or sensitivity analyses. A fixed effects model was used when heterogeneity was low and studies could be combined. When a single study had a large weight, sensitivity analysis was performed to check stability. When substantial heterogeneity remained unexplained, meta-analysis was not performed and a descriptive analysis was provided instead.

#### 2.5.4 Subgroup and meta-regression analyses

To explore heterogeneity, we pre-specified subgroup and meta-regression analyses according to acupuncture methods (manual or specific), whether exercise modalities varied by NYHA class, number of acupoints (< 5 or ≥5), treatment duration (< 4 weeks or ≥4 weeks), and concomitant HF medication (reported or not). Subgroup analyses were conducted when at least two studies were available for each category. For outcomes with significant heterogeneity, meta-regression examined the association between covariates and effect sizes. All analyses used R 4.1.

#### 2.5.5 Sensitivity analyses and publication bias

Sensitivity analyses included sequential exclusion of individual studies and exclusion of studies at high risk of bias. Because fewer than 10 studies were available for each outcome, publication bias could not be reliably assessed. Doi plots and LFK indices were therefore reported for transparency only and interpreted with extreme caution (19).

## 3 Results

### 3.1 Literature search

**Figure 1:**
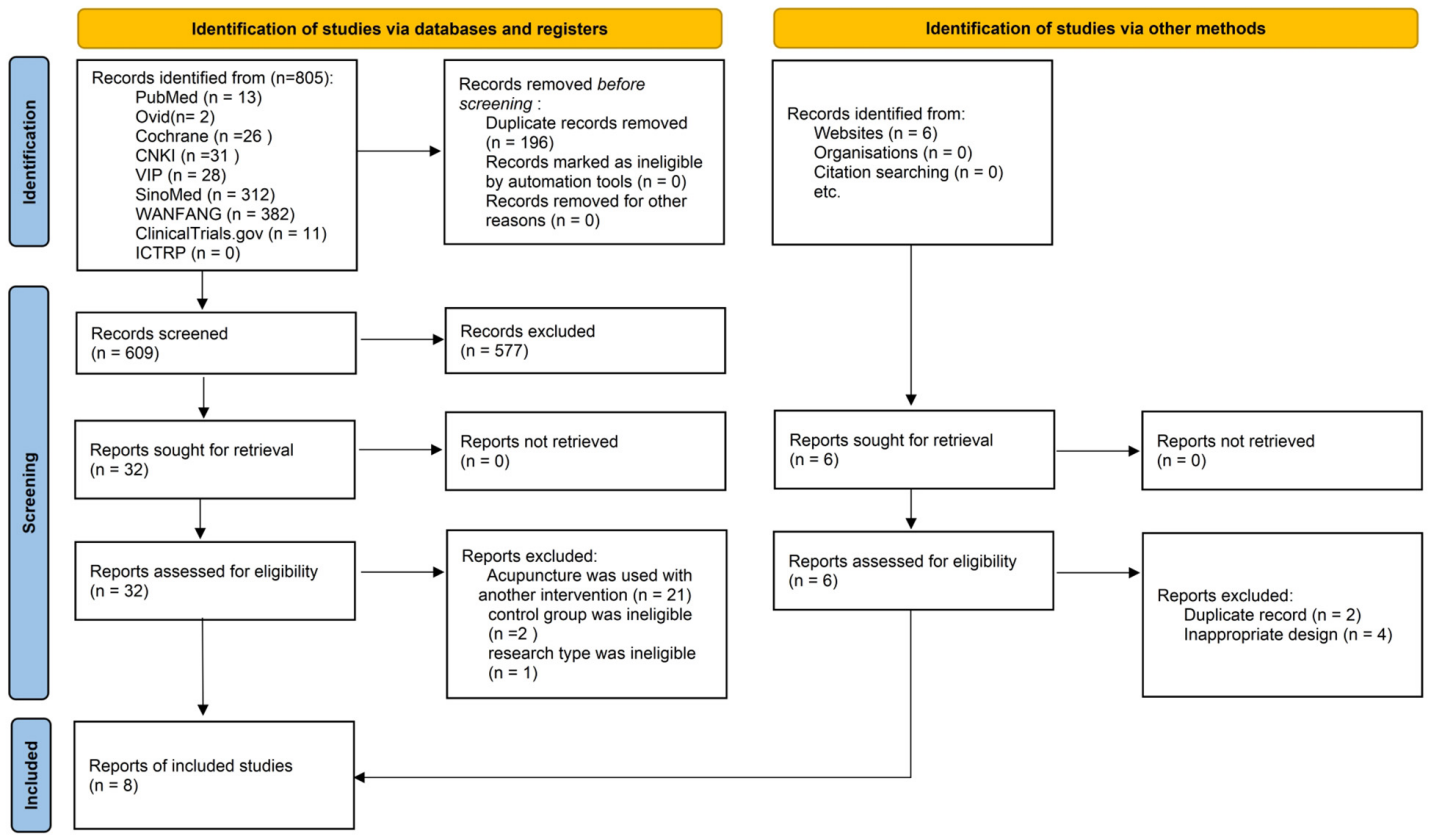
PRISMA flow diagram (https://www.prisma-statement.org/prisma-2020-flow-diagram).

A total of 805 records were identified through database searching. After removing 196 duplicates with NoteExpress, 609 articles remained. Two independent reviewers screened titles and abstracts and excluded 577 irrelevant records, with an inter-rater reliability Kappa statistic (K = 0.797). The remaining 32 full-text articles were assessed. Of these, 24 were excluded for the following reasons: acupuncture combined with other interventions (*n* = 21), ineligible control intervention (*n* = 2), or inappropriate study design (*n* = 1). Finally, eight RCTs were included. Seven were published in Chinese journals and one in English, with publication years ranging from 2021 to 2025. Through other methods, six records were identified from websites. All were assessed, resulting in the exclusion of two duplicates and four studies with unsuitable designs. No additional unpublished or ongoing studies were identified through trial registries. The eight included studies involved 668 participants. Supplementary Table S3 lists the excluded studies and reasons for exclusion. The screening process is shown in Figure 1, and study characteristics in Tables 1, 2.

**Table 1:** Study characteristics.

Country of study	Authors, year	Age mean (SD)		Sample size		Intervention		NYHA	Outcomes
		T	C	T	C	T	C		
China	Fu et al. (25)	66.75 (3.52)	66.82 (3.69)	48	48	WN+ER	ER	II, III	①②④⑤
China	Zheng et al. (24)	64.51 (4.50)	64.25 (4.47)	40	40	MA + ER + Baduanjin	ER	I–III	①②③
China	Lai et al. (23)	51.87 (5.82)	51.59 (5.78)	47	47	WN + ER + RT	ER + RT	II, III	①②③④⑤
China	Fan et al. (27)	66.10 (1.98)	65.36 (2.14)	31	31	PN + ER + RT	ER + RT	II, III	①②③
China	Tian et al. (21)	52.82 (7.45)	53.16 (7.14)	57	57	MA + ER + RT	ER + RT	II, III	①②③④⑤
China	Ren et al. (26)	68.58 (6.07)	67.09 (6.34)	38	35	MA + ER + RT	ER + RT	II, III	①
China	Wu et al. (22)	60.78 (7.45)	59.24 (8.20)	35	34	MA + ER + RT	ER + RT	II, III	①③⑤
Serbia	Ilic et al. (20)	73.5 (10.5)^1^	76 (12.7)^1^	40	40	MA + ER + RT	ER + RT	II, III	②

MA, manual acupuncture; EA, electroacupuncture; WN, warming needle moxibustion; PN, press-needle; ER, exercise rehabilitation; RT, Routine treatment; 1, median (IQR); ①, LVEF; ②, 6MWD; ③, MLHFQ; ④, LVESD; and ⑤, LVEDD.

**Table 2:** Details of the acupuncture and exercise interventions.

Authors, year	Acupuncture protocol	ER protocol	Period
Fu et al. (25)	Bil (BL13, BL14, BL15); 25 min; once daily.	NYHA II: walking, stair climbing, and Tai Chi; NYHA III: walking, stair climbing, and joint movement	8 weeks
Zheng et al. (24)	Uk (BL15, PC6, CV14, CV17); 20 min; once daily; five times weekly.	Baduanjin, aerobic exercise, resistance training, and flexibility training	8 weeks
Lai et al. (23)	Bil (SP10, PC6, ST40, ST36); 25 min; once daily; six times weekly.	Stationary bike training, walking, and medical gymnastics	4 weeks
Fan et al. (27)	Bil (PC6, PC4, PC3, SP10), and CV4; 24 h; once daily; five times weekly.	Resistance training with elastic bands	8 weeks
Tian et al. (21)	Uk (PC6, SP10, ST40, ST36, HT7, BL15); 30 min; once daily.	Walking, aerobics, and stationary bike training	4 weeks
Ren et al. (26)	Bil (PC6, BL15, ST36, CV17, HT7, SP10, PC4); 30 min; once daily; five times weekly.	Aerobic exercise primarily involving limb training	8 weeks
Wu et al. (22)	Bil (CV6, PC6, PC4, KI7, KI9, HT7, SP6); 30 min; once daily; five times weekly.	Warm-up exercises and walking	4 weeks
Ilic et al. (20)	Uk (PC6, HT7, LI4, SP9, ST36), GV20, and CV17; 20 min; once daily	Breathing techniques, limb cycle training, and arm/leg exercises	5 to 10 days

Bil, bilateral; Uk, unknown; BL13, Feishu acupoint; BL14, Jueyinshu acupoint; BL15, Xinshu acupoint; PC3, Quze acupoint; PC4, Ximen acupoint; PC6, Neiguan acupoint; CV4, Guanyuan acupoint; CV6, Qihai acupoint; CV14, Juque acupoint; CV17, Danzhong acupoint; SP10, Xuehai acupoint; ST40, Fenglong acupoint; ST36, Zusanli acupoint; HT7, Shenmen acupoint; KI7, Fuliu acupoint; KI9, Zhubin acupoint; SP6, Sanyinjiao acupoint; LI4, Hegu acupoint; SP9, Yinlingquan acupoint; and GV20, Baihui acupoint.

### 3.2 Quality assessment of included studies

**Figure 2:**
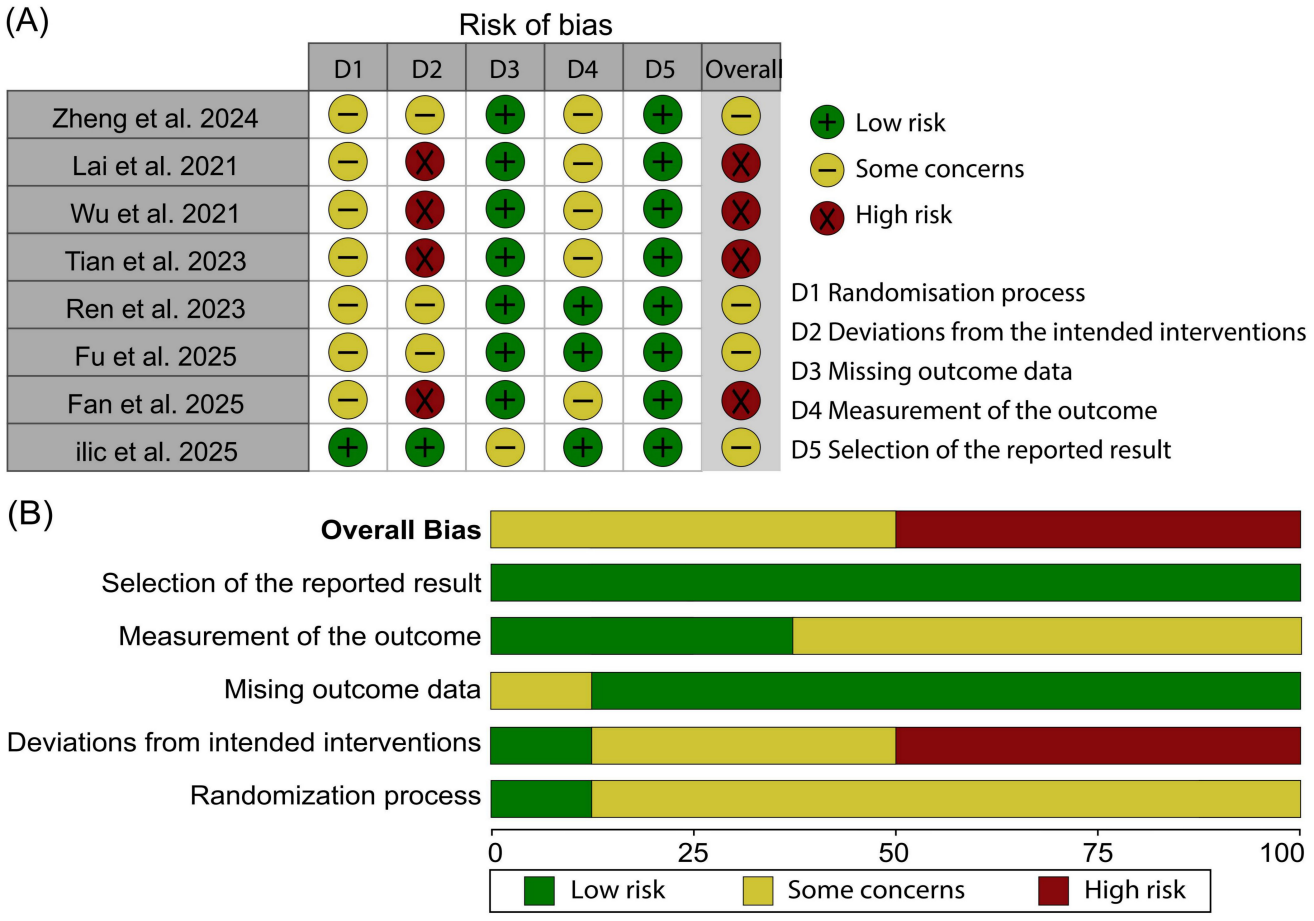
The risk of bias of the included studies **(A)** Risk of bias graph. **(B)** Risk of bias summary.

Two independent researchers evaluated eight articles using the RoB 2 tool, with the inter-rater reliability based on the kappa statistic. (1) Randomization process: Only one study (20) was rated as low risk; seven (21–27) had some concerns (randomization mentioned but no allocation concealment). (2) Deviations from intended interventions: Blinding of practitioners is inherently difficult in acupuncture trials, so single-blinding is the only feasible option. One study (20) had low risk; three were judged as some concerns (24–26) (unclear blinding but objective outcomes); the remaining included subjective outcomes highly susceptible to knowledge of group allocation, and were rated as high risk. (3) Missing outcome data: Ilic et al. (20) reported 21 dropouts (87.5 to 95.0% missing data), likely affecting robustness, thus rated as some concerns; the remaining studies were low risk. (4) Measurement of the outcome: The results of the MLHFQ score may be influenced by subjective factors; therefore, five studies (21–24, 27) were rated as having some concerns. (5) selection of the reported result: All studies had no selective outcome reporting and were therefore rated as low risk. (6) Overall bias: The overall risk of bias was mixed: four studies was rated as some concerns, and four had high risk. Main issues included lack of allocation concealment and blinding. (Figure 2).

### 3.3 Outcome indicators

#### 3.3.1 LVEF

**Figure 3:**
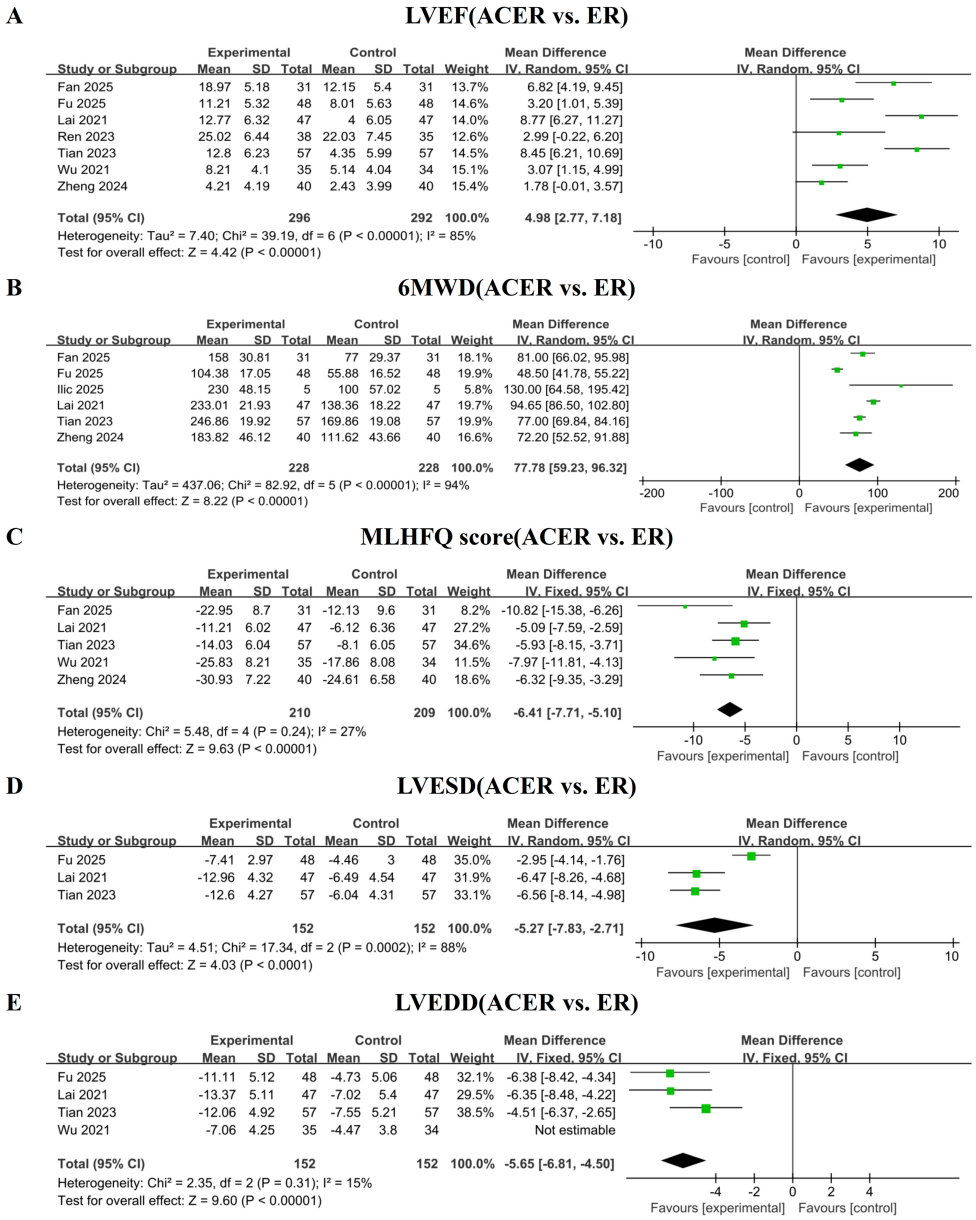
The forest plots comparing acupuncture combined with exercise rehabilitation (ACER) vs. exercise rehabilitation (ER) alone for heart failure patients. **(A)** Left ventricular ejection fraction (LVEF, %). **(B)** Six-min walk distance (6MWD, meters). **(C)** Minnesota Living with Heart Failure Questionnaire (MLHFQ) score. **(D)** Left ventricular end-systolic diameter (LVESD, mm). **(E)** Left ventricular end-diastolic diameter (LVEDD, mm).

Seven studies reported the effect of the ACER group compared with the ER group on improving LVEF, with 588 patients (296 in ACER and 292 in ER). Heterogeneity was substantial (*P*<*0.00001*, *I*^2^ = 85%). To assess the robustness of the pooled estimate, we performed sensitivity analyses by excluding the studies with the highest risk and further excluding each study sequentially, but substantial heterogeneity persisted. Subgroup analyses were conducted according to treatment duration, acupuncture modality, number of acupoints, acupuncture frequency, and concomitant conventional HF medication use, but none explained the heterogeneity. Subgroup analysis by exercise type was not feasible because of diversity of exercise protocols. A random-effects model showed that ACER was associated with greater improvement in LVEF than ER alone (*MD* = 4.98, 95% CI [2.77, 7.18], *P* < 0.00001), as shown in Figure 3A. Given the substantial unexplained heterogeneity, this result should be interpreted with caution.

#### 3.3.2 6MWD

Six studies reported the effect of the ACER group compared with the ER group on improving 6MWD, with 456 patients (228 in each group). Heterogeneity was substantial (*P*<*0.00001*, *I*^2^ = 94%). Sensitivity analyses did not resolve the heterogeneity. Subgroup analyses based on treatment duration, acupuncture modality, acupoint number, and acupuncture frequency were performed. Although partial subgroups (manual acupuncture, ≥5 acupoints, five times per week) showed low internal heterogeneity, no significant inter-subgroup differences were observed, indicating that these factors did not explain the overall high heterogeneity. Subgroup analyses by exercise type and baseline medication were not conducted because of the small number of studies and inconsistent reporting. A random-effects model showed that ACER was associated with greater improvement in 6MWD than ER alone (*MD* = 77.78 m, 95% CI [59.23, 96.32], *P* < 0.00001), as shown in Figure 3B. Given the substantial unexplained heterogeneity and potential bias from sparse data, the result is not robust.

#### 3.3.3 MLHFQ score

Five studies reported the effect of the ACER group compared with the ER group on reducing MLHFQ score, with 419 patients (210 in ACER and 209 in ER). Heterogeneity was low (*P* = *0.24*, *I*^2^ = 27%). A fixed-effects model showed that ACER was associated with a greater reduction in MLHFQ score than ER alone (*MD* = −6.41, 95% CI [−7.71, −5.10], *P* < 0.00001), as shown in Figure 3C.

#### 3.3.4 LVESD

Three studies reported the effect of the ACER group compared with the ER group on reducing LVESD, with 304 patients (152 in each group). Significant heterogeneity was observed (*P* = *0.0002*, *I*^2^ = 88%). A random-effects model showed that ACER was associated with a greater reduction in LVESD than ER alone (*MD* = −5.27 mm, 95% CI [−7.83, −2.71], *P* < 0.00001), as shown in Figure 3D. Only three studies reported LVESD, precluding meaningful subgroup or sensitivity analyses. Accordingly, this pooled estimate should be interpreted with considerable caution.

#### 3.3.5 LVEDD

Four studies reported the effect of the ACER group compared with the ER group on reducing LVEDD, with 373 patients (187 in ACER and 186 in ER). Heterogeneity was substantial (*P* = *0.02*, *I*^2^ = 69%). Further sensitivity analyses were performed by sequentially excluding each study. After removing one study, heterogeneity decreased markedly (*P* = 0.31, *I*^2^ = 15%). A fixed-effects model showed that ACER was associated with a greater reduction in LVEDD than ER alone (*MD* = −5.65 mm, 95% CI [−6.81, −4.50], *P* < 0.00001), as shown in Figure 3E.

### 3.6 Exploratory meta-regression analysis

These analyses were underpowered (k = 8) and prone to type I and type II errors. Therefore, no explanatory or predictive conclusions can be drawn. Exploratory univariate meta-regression did not provide a robust explanation for the heterogeneity observed in LVEF or 6MWD. Although NYHA-based exercise stratification reached nominal significance for 6MWD, this finding is unstable because the evidence base was small and most covariate categories contained only one or two studies. We therefore retained these results as descriptive information only and did not use them to support any definitive conclusions (Supplementary Table S4).

### 3.7 Publication bias

**Figure 4:**
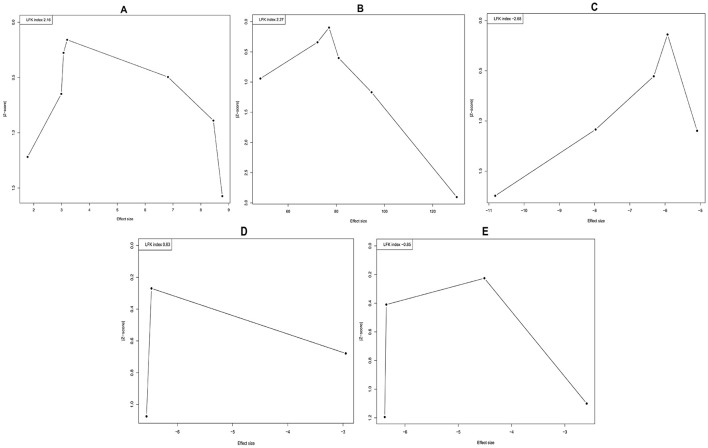
Doi plots for assessment of publication bias. Reported LFK indices: **(A)** Left ventricular ejection fraction (LVEF = 2.16). **(B)** 6-min walk distance (6MWD = 2.27). **(C)** Minnesota Living with Heart Failure Questionnaire (MLHFQ = −2.68). **(D)** Left ventricular end-systolic diameter (LVESD = 0.83). **(E)** Left ventricular end-diastolic diameter (LVEDD = −0.85).

Because all outcomes were informed by fewer than 10 studies, publication bias could not be reliably assessed. Doi plots and LFK indices were retained only for transparency and should be interpreted with extreme caution rather than as confirmatory tests (19). The reported LFK indices were 2.156 for LVEF, 2.267 for 6MWD, −2.681 for MLHFQ, 0.833 for LVESD, and −0.851 for LVEDD (Figure 4; Supplementary Table S5). Given the limited evidence base and the pre-dominance of Chinese-language studies, these values do not provide firm evidence for or against publication bias.

### 3.8 GRADE evidence quality classification

The quality of evidence for all outcomes was evaluated using the GRADE guidelines. LVEF, 6MWD, MLHFQ, and LVESD were rated as very low certainty because of combined concerns regarding risk of bias, heterogeneity, imprecision, and exploratory publication-bias signals. LVEDD was rated as low certainty. Outcome-specific downgrading reasons are summarized in Table 3.

**Table 3:** GRADE evidence profiles and summary of findings table.

Outcome (studies)	No. of participants		Risk of bias	Inconsistency	Indirectness	Imprecision	Other considerations	Publication bias	Overall certainty of evidence	Anticipated absolute effects (95% CI)
	ACER	ER								
LVEF (7 RCTs)	296	292	Serious^a^	Very serious^b^	Not serious	Serious^d^	None	Serious^e^	⊕°°° Very low	*MD* 4.98 % higher (2.77 higher to 7.18 higher)
6MWD (6 RCTs)	228	228	Serious^a^	Very serious^b^	Not serious	Serious^d^	None	Serious^e^	⊕°°° Very Low	*MD* 77.78 m higher (59.23 higher to 96.32 higher)
MLHFQ (5 RCTs)	210	209	Serious^a^	Not serious	Not serious	Serious^d^	None	Serious^e^	⊕°°° Very Low	*MD* 6.41 points lower (7.71 lower to 5.10 lower)
LVESD (3 RCTs)	152	152	Serious^a^	Very serious^b^	Not serious	Very serious^d^	None	None	⊕°°° Very low	*MD* 5.27 mm lower (7.83 lower to 2.71 lower)
LVEDD (4 RCTs)	187	186	Serious^a^	Serious^c^	Not serious	Serious^d^	None	None	⊕⊕°°Low	*MD* 5.65 mm lower (6.81 lower to 4.50 lower)

^a^Risk of bias serious because 7/8 studies lacked allocation concealment and blinding was limited. ^b^Inconsistency very serious because LVEF, 6MWD, and LVESD showed marked heterogeneity (I^2^ = 85, 94, and 88%, respectively). ^c^LVEDD retained moderate heterogeneity (I^2^ = 69%). ^d^Imprecision serious/very serious because pooled estimates were based on few studies and modest sample sizes. ^e^An assessment of publication bias indicated that the LFK indices for LVEF, 6MWD, and MLHFQ all exceeded two.

## 4 Discussion

### 4.1 Summary of the main findings

This meta-analysis of eight RCTs identified statistically significant pooled differences favoring ACER for LVEF, 6MWD, MLHFQ, LVESD, and LVEDD. However, the evidence base is fragile: most studies had methodological limitations, between-study heterogeneity was substantial for several outcomes, and the GRADE certainty of evidence ranged from low to very low. The current results therefore are still insufficient to establish reliable evidence of clinical benefit.

The observed 4.98% improvement in LVEF is difficult to interpret clinically because the estimate is supported by very low-certainty evidence, high unexplained heterogeneity, and weak trial methodology. The 77.78-meter improvement in 6MWD exceeds commonly cited minimal clinically important differences, but the magnitude is likely inflated by bias, heterogeneity, and sparse data. By comparison, reductions in LVEDD and MLHFQ were more consistent, yet MLHFQ was still downgraded to very low certainty because of potential publication bias, and the lack of long-term follow-up limits clinical interpretation. Their clinical significance therefore remains uncertain.

### 4.2 Therapeutic mechanisms and evidence gap of acupuncture

The eight studies involved a total of 20 acupoints. The most frequently used were PC6 (Neiguan, 7/8 studies), BL15 (Xinshu, 4/8), ST36 (Zusanli, 4/8), and HT7 (Shenmen, 4/8). Existing animal studies suggest that these acupoints may influence cardiac autonomic function, inflammatory signaling, and ventricular remodeling (28–32), which provides biological plausibility for adjunctive acupuncture in HF. However, none of the included RCTs directly measured relevant biomarkers. Therefore, the clinical relevance of these proposed mechanisms remains unsubstantiated in the context of ACER for HF. Future trials should incorporate relevant biomarkers so that observed clinical effects can be linked to verifiable biological changes.

### 4.3 Clinical feasibility and implementation considerations

An important practical issue is the intensity of the intervention itself. As shown in Table 2, most acupuncture protocols required daily or near-daily sessions for 4 to 8 weeks, in addition to structured exercise rehabilitation. For patients with HF, particularly older adults and those with functional limitation, this protocol may impose substantial travel, time, financial, and physical burdens. Such demands could reduce patient adherence and limit scalability in practical applications. Future trials should therefore report attendance, treatment completion, direct and indirect costs, and patient acceptability to better evaluate the real-world feasibility of ACER.

### 4.4 Limitations

This review has several major limitations. (1) Substantial risk of bias: only one study reported allocation concealment using sealed envelopes, and only one implemented blinding. This is particularly problematic for subjective outcomes such as MLHFQ, where knowledge of group assignment may have inflated the apparent benefit. (2) Limited evidence base and potential publication bias: despite a comprehensive search, only eight RCTs were included, seven from China and one from Serbia. The limited number and geographical concentration of studies increase the risk of publication bias and imprecision. The only non-Chinese study also provided limited reference value because of missing data. (3) Unexplained high heterogeneity: marked variation was present across acupuncture protocols, exercise prescriptions, treatment durations, and concomitant therapies. Although subgroup and meta-regression analyses were attempted, they lacked sufficient statistical power and could not provide a robust explanation for the observed inconsistency. (4) Absence of safety data and long-term follow-up: all included studies focused on short-term outcomes, and none systematically reported adverse events. This prevents a balanced evaluation of the risk-benefit profile of ACER. Collectively, these limitations reduce confidence in the pooled estimates, and no reliable conclusion can yet be drawn regarding the actual clinical value of ACER for HF.

### 4.5 Recommendations for future trials

Future trials should adopt rigorous randomization with adequate allocation concealment, use sham acupuncture or other credible control procedures, and ensure blinded outcome assessment. Prospective registration, a sample-size calculation, standardized intervention protocols, and adverse-event monitoring are essential. Trials should systematically collect acupuncture-related adverse events (e.g., bleeding, pain, infection) and exercise-related harms (e.g., falls or symptom exacerbation), while incorporating mechanistic biomarkers and adherence data, to elucidate their clinical feasibility.

## 5 Conclusion

ACER was associated with favorable effects on several short-term outcomes in patients with HF. However, the certainty of evidence was low to very low. Given the substantial risk of bias, heterogeneity, imprecision, uncertain publication bias, and lack of safety data, the current evidence is insufficient to support a definitive clinical recommendation. ACER should be regarded as a promising but unproven adjunctive strategy. Further well-designed RCTs are needed to clarify the efficacy and safety in HF rehabilitation.

## Data Availability

The raw data supporting the conclusions of this article will be made available by the authors, without undue reservation.

## Supplementary material

Supplementary Table S1 PRISMA 2020 checklist.

Supplementary Table S2 Search strategy.

Supplementary Table S3 Excluded studies and the reasons for exclusion.

Supplementary Table S4 Meta-regression analyses.

Supplementary Table S5 LFK details and interpretation for each outcome.

Supplementary Appendix S1 GRADE evidence evaluation criteria.
