# Supplementary material for: Efficacy of acupuncture combined with exercise rehabilitation on cardiac function in patients with heart failure: a systematic review and meta-analysis
Source: Front Med (Lausanne). 2026 Jul 2;13:1789189. doi: 10.3389/fmed.2026.1789189 (PMC13374047; doi:10.3389/fmed.2026.1789189)
Supplement: Supplementary Table S1 — PRISMA 2020 checklist. [file Table_1.DOCX]

***Supplementary Material***

**Supplemental Table S1**. Preferred Reporting Items for Systematic Reviews and Meta-Analyses 2020 (PRISMA 2020) Checklists

PRISMA 2020 Main Checklist

| **Topic** | **No.** | **Item** | **Location where item is reported** |
| --- | --- | --- | --- |
| **TITLE** |  |  |  |
| **Title** | 1 | Identify the report as a systematic review. | Title |
| **ABSTRACT** |  |  |  |
| **Abstract** | 2 | See the PRISMA 2020 for Abstracts checklist. | Methods |
| **INTRODUCTION** |  |  |  |
| **Rationale** | 3 | Describe the rationale for the review in the context of existing knowledge. | Introduction |
| **Objectives** | 4 | Provide an explicit statement of the objective(s) or question(s) the review addresses. | Introduction |
| **METHODS** |  |  |  |
| **Eligibility criteria** | 5 | Specify the inclusion and exclusion criteria for the review and how studies were grouped for the syntheses. | Eligibility criteria |
| **Information sources** | 6 | Specify all databases, registers, websites, organisations, reference lists and other sources searched or consulted to identify studies. Specify the date when each source was last searched or consulted. | Search strategy;  Supplemental Table S2 |
| **Search strategy** | 7 | Present the full search strategies for all databases, registers and websites, including any filters and limits used. | Search strategy;  Supplemental Table S2 |
| **Selection process** | 8 | Specify the methods used to decide whether a study met the inclusion criteria of the review, including how many reviewers screened each record and each report retrieved, whether they worked independently, and if applicable, details of automation tools used in the process. | Literature search;  Search strategy |
| **Data collection process** | 9 | Specify the methods used to collect data from reports, including how many reviewers collected data from each report, whether they worked independently, any processes for obtaining or confirming data from study investigators, and if applicable, details of automation tools used in the process. | Literature search |
| **Data items** | 10a | List and define all outcomes for which data were sought. Specify whether all results that were compatible with each outcome domain in each study were sought (e.g. for all measures, time points, analyses), and if not, the methods used to decide which results to collect. | Eligibility criteria |
|  | 10b | List and define all other variables for which data were sought (e.g. participant and intervention characteristics, funding sources). Describe any assumptions made about any missing or unclear information. | Eligibility criteria |
| **Study risk of bias assessment** | 11 | Specify the methods used to assess risk of bias in the included studies, including details of the tool(s) used, how many reviewers assessed each study and whether they worked independently, and if applicable, details of automation tools used in the process. | Data extraction and quality assessment |
| **Effect measures** | 12 | Specify for each outcome the effect measure(s) (e.g. risk ratio, mean difference) used in the synthesis or presentation of results. | Statistical analysis |
| **Synthesis methods** | 13a | Describe the processes used to decide which studies were eligible for each synthesis (e.g. tabulating the study intervention characteristics and comparing against the planned groups for each synthesis (item 5)). | Statistical analysis |
|  | 13b | Describe any methods required to prepare the data for presentation or synthesis, such as handling of missing summary statistics, or data conversions. | Statistical analysis |
|  | 13c | Describe any methods used to tabulate or visually display results of individual studies and syntheses. | Statistical analysis |
|  | 13d | Describe any methods used to synthesize results and provide a rationale for the choice(s). If meta-analysis was performed, describe the model(s), method(s) to identify the presence and extent of statistical heterogeneity, and software package(s) used. | Statistical analysis |
|  | 13e | Describe any methods used to explore possible causes of heterogeneity among study results (e.g. subgroup analysis, meta-regression). | Statistical analysis |
|  | 13f | Describe any sensitivity analyses conducted to assess robustness of the synthesized results. | Statistical analysis |
| **Reporting bias assessment** | 14 | Describe any methods used to assess risk of bias due to missing results in a synthesis (arising from reporting biases). | Statistical analysis |
| **Certainty assessment** | 15 | Describe any methods used to assess certainty (or confidence) in the body of evidence for an outcome. | Statistical analysis |
| **RESULTS** |  |  |  |
| **Study selection** | 16a | Describe the results of the search and selection process, from the number of records identified in the search to the number of studies included in the review, ideally using a flow diagram. | Figure 1 |
|  | 16b | Cite studies that might appear to meet the inclusion criteria, but which were excluded, and explain why they were excluded. | Figure 1;  Table S3 |
| **Study characteristics** | 17 | Cite each included study and present its characteristics. | Table 1 |
| **Risk of bias in studies** | 18 | Present assessments of risk of bias for each included study. | Figure 2 |
| **Results of individual studies** | 19 | For all outcomes, present, for each study: (a) summary statistics for each group (where appropriate) and (b) an effect estimate and its precision (e.g. confidence/credible interval), ideally using structured tables or plots. | Figure 3 |
| **Results of syntheses** | 20a | For each synthesis, briefly summarise the characteristics and risk of bias among contributing studies. | Quality assessment of included studies |
|  | 20b | Present results of all statistical syntheses conducted. If meta-analysis was done, present for each the summary estimate and its precision (e.g. confidence/credible interval) and measures of statistical heterogeneity. If comparing groups, describe the direction of the effect. | Outcome indicators |
|  | 20c | Present results of all investigations of possible causes of heterogeneity among study results. | Outcome indicators |
|  | 20d | Present results of all sensitivity analyses conducted to assess the robustness of the synthesized results. | Outcome indicators |
| **Reporting biases** | 21 | Present assessments of risk of bias due to missing results (arising from reporting biases) for each synthesis assessed. | Outcome indicators |
| **Certainty of evidence** | 22 | Present assessments of certainty (or confidence) in the body of evidence for each outcome assessed. | Table 3 |
| **DISCUSSION** |  |  |  |
| **Discussion** | 23a | Provide a general interpretation of the results in the context of other evidence. | Summary of the main findings |
|  | 23b | Discuss any limitations of the evidence included in the review. | Limitations |
|  | 23c | Discuss any limitations of the review processes used. | Limitations |
|  | 23d | Discuss implications of the results for practice, policy, and future research. | Conclusion |
| **OTHER INFORMATION** |  |  |  |
| **Registration and protocol** | 24a | Provide registration information for the review, including register name and registration number, or state that the review was not registered. | Protocol and registration |
|  | 24b | Indicate where the review protocol can be accessed, or state that a protocol was not prepared. | Protocol and registration |
|  | 24c | Describe and explain any amendments to information provided at registration or in the protocol. | Not applicable |
| **Support** | 25 | Describe sources of financial or non-financial support for the review, and the role of the funders or sponsors in the review. | Funding |
| **Competing interests** | 26 | Declare any competing interests of review authors. | Conflicts of interest |
| **Availability of data, code and other materials** | 27 | Report which of the following are publicly available and where they can be found: template data collection forms; data extracted from included studies; data used for all analyses; analytic code; any other materials used in the review. | Supplementary material |

**PRIMSA Abstract Checklist**

| **Topic** | **No.** | **Item** | **Reported?** |
| --- | --- | --- | --- |
| **TITLE** |  |  |  |
| **Title** | 1 | Identify the report as a systematic review. | Yes |
| **BACKGROUND** |  |  |  |
| **Objectives** | 2 | Provide an explicit statement of the main objective(s) or question(s) the review addresses. | Yes |
| **METHODS** |  |  |  |
| **Eligibility criteria** | 3 | Specify the inclusion and exclusion criteria for the review. | Yes |
| **Information sources** | 4 | Specify the information sources (e.g. databases, registers) used to identify studies and the date when each was last searched. | Yes |
| **Risk of bias** | 5 | Specify the methods used to assess risk of bias in the included studies. | Yes |
| **Synthesis of results** | 6 | Specify the methods used to present and synthesize results. | Yes |
| **RESULTS** |  |  |  |
| **Included studies** | 7 | Give the total number of included studies and participants and summarise relevant characteristics of studies. | Yes |
| **Synthesis of results** | 8 | Present results for main outcomes, preferably indicating the number of included studies and participants for each. If meta-analysis was done, report the summary estimate and confidence/credible interval. If comparing groups, indicate the direction of the effect (i.e. which group is favoured). | Yes |
| **DISCUSSION** |  |  |  |
| **Limitations of evidence** | 9 | Provide a brief summary of the limitations of the evidence included in the review (e.g. study risk of bias, inconsistency and imprecision). | Yes |
| **Interpretation** | 10 | Provide a general interpretation of the results and important implications. | Yes |
| **OTHER** |  |  |  |
| **Funding** | 11 | Specify the primary source of funding for the review. | Yes |
| **Registration** | 12 | Provide the register name and registration number. | Yes |

*From:* Page MJ, McKenzie JE, Bossuyt PM, Boutron I, Hoffmann TC, Mulrow CD, et al. The PRISMA 2020 statement: an updated guideline for reporting systematic reviews. MetaArXiv. 2020, September 14. DOI: 10.31222/osf.io/v7gm2. For more information, visit: [www.prisma-statement.org](file:///C:\\Users\\ssk\\Downloads\\www.prisma-statement.org)

**Supplementary Table S2.** Search strategy

| **Database** | PubMed |
| --- | --- |
| **Search Terms** | Title/Abstract (Acupuncture OR electroacupuncture OR acupoint) AND (Heart Failure OR cardiac failure OR ventricular dysfunction OR myocardial failure) AND (Exercise OR physical activity OR sports OR training OR Taiji OR Baduanjin) |

| **Database** | Ovid |
| --- | --- |
| **Search Terms** | ((Acupuncture or electroacupuncture or acupoint) and (Heart Failure or cardiac failure or ventricular dysfunction or myocardial failure) and (Exercise or physical activity or sports or training or Taiji or Baduanjin)).ab. |

| **Database** | Cochrane Library |
| --- | --- |
| **Search Terms** | Acupuncture OR electroacupuncture OR acupoint in Title Abstract Keyword AND Heart Failure OR cardiac failure OR ventricular dysfunction OR myocardial failure in Title Abstract Keyword AND Exercise OR physical activity OR sports OR training OR Taiji OR Baduanjin in Title Abstract Keyword |

| **Database** | [ClinicalTrials.gov](https://clinicaltrials.gov/) |
| --- | --- |
| **Search Terms** | (Heart Failure OR cardiac failure OR ventricular dysfunction OR myocardial failure) AND (Acupuncture OR electroacupuncture OR acupoint) AND (Exercise OR physical activity OR sports OR training OR Taiji OR Baduanjin) |

| **Database** | ICTRP |
| --- | --- |
| **Search Terms** | (Heart Failure OR cardiac failure OR ventricular dysfunction OR myocardial failure) AND (Acupuncture OR electroacupuncture OR acupoint) AND (Exercise OR physical activity OR sports OR training OR Taiji OR Baduanjin) |

| **Database** | China National Knowledge Infrastructure |
| --- | --- |
| **Search Terms** | (心力衰竭 + 心衰 + HF + CHF + 心功能) AND (针 + 针刺 + 针灸 +电针 + 火针 + 腧穴) AND (运动康复 + 心脏康复 + 训练 + 锻炼 + 八段锦 + 太极拳) AND (随机对照试验 + 试验 + 随机 + 临床试验 +对照 + 临床研究)(Title/Abstract/Keyword) |

| **Database** | Weipu Database |
| --- | --- |
| **Search Terms** | (心力衰竭 OR 心衰 OR CHF OR HF OR 心功能) AND (针 OR 针灸 OR 针刺 OR 电针 OR 火针 OR 腧穴) AND (运动康复 OR 心脏康复 OR 训练 OR 锻炼 OR 八段锦 OR 太极拳) AND (随机对照试验 OR 试验 OR 随机 OR 对照 OR 临床研究)(Title/Abstract/Keyword) |

| **Database** | Sinomed Database |
| --- | --- |
| **Search Terms** | ( "心力衰竭"[全部字段] OR "心衰"[全部字段] OR "CHF"[全部字段] OR "HF"[全部字段] OR "心功能"[全部字段]) AND ( "针"[全部字段] "针灸"[全部字段] OR "针刺"[全部字段] OR "电针"[全部字段] OR "火针"[全部字段] OR "腧穴"[全部字段]) AND ( "运动康复"[全部字段] OR "心脏康复"[全部字段] OR "训练"[全部字段] OR "锻炼"[全部字段] OR "八段锦"[全部字段] OR "太极拳"[全部字段]) AND ( "随机对照试验"[全部字段] OR "随机"[全部字段] OR "对照"[全部字段] OR "试验"[全部字段] OR "临床研究"[全部字段]) |

| **Database** | WANFANG Medical |
| --- | --- |
| **Search Terms** | (心力衰竭 OR 心衰 OR CHF OR HF OR 心功能) AND (针 OR 针灸 OR 针刺 OR 电针 OR 火针 OR 腧穴) AND (运动康复 OR 心脏康复 OR 训练 OR 锻炼 OR 八段锦 OR 太极拳) AND (随机对照试验 OR 试验 OR 随机 OR 对照 OR 临床研究) (主题) |

**Supplementary Table 3.** Excluded studies and the reason for exclusion

| Study  （in Chinese） | Study  （in English） | Reason for exclusion |
| --- | --- | --- |
| 刘昕, 黎明华. 针灸联合八段锦对冠心病心力衰竭患者运动康复及生活质量的影响[J]. 中外医学研究, 2024, 22(4): 1-4. | Effect of Acupuncture Combined with Baduanjin on Exercise Rehabilitation and Quality of Life in Patients with Coronary Heart Disease and Heart Failure | control group was ineligible |
| 柯俊华,邱福山,范文曦,等. 针刺结合有氧运动对慢性心力衰竭患者心肺储备能力的影响[J]. 中国卫生标准管理,2022,13(1):107-110. | Effect of Acupuncture Combined With Aerobic Exercise Training on Cardiopulmonary Reserve Function in Patients With Chronic Heart Failure | control group was ineligible |
| 郭雪峰,成丽娟,刘桂林.针刺足三里及丰隆治疗顽固性心力衰竭30例临床观察[J].山东中医药大学学报,2020,44(03):282-285. | Clinical Observation on Acupuncture at Zusanli and Fenglong in Treating 30 Patients with Refractory Heart Failure | Intervention was ineligible |
| 贺卫,朱慧英,丁嘉怡,等.中药联合针刺治疗气虚血瘀证慢性心力衰竭（心功能分级Ⅱ级）的临床疗效[J].上海中医药大学学报,2022,36(S1):76-79+83. | Clinical efficacy of traditional Chinese medicine Combined with acupuncture on chronic heart failure (cardiac function grade Ⅱ) with Qi deficiency and blood stasis syndrome | Intervention was ineligible |
| 邱福山,范文曦,杨如杏,等.揿针联合常规抗心衰方案治疗阳气亏虚血瘀型慢性心力衰竭30例[J].福建中医药,  2023,54(09):60-63. | Treatment of 30 Cases of Chronic Heart Failure of Yang Qi Deficiency and Blood Stasis Type with Thumbtack Needle Acupuncture Combined with Conventional Anti-Heart Failure Regimen | Intervention was ineligible |
| 贺卫,朱慧英,丁嘉怡,等.中药联合针刺治疗气虚血瘀证慢性心力衰竭（心功能分级Ⅱ级）的临床疗效[J].上海中医药大学学报,2022,36(S1):76-79+83. | Clinical efficacy of traditional Chinese medicine combined with acupuncture on chronic heart failure (cardiac function grade II) with Qi deficiency and blood stasis syndrome | Intervention was ineligible |
| 于大印.针药联合运动康复训练对冠心病心力衰竭患者的影响[J].实用中西医结合临床,2022,22(08):14-16+20. | Influence of Acupuncture and Medicine Combined with Exercise Rehabilitation Training on Patients with Coronary Heart Disease and Heart Failure | Intervention was complicated |
| 李婷,苏士印,康广山,等.益气健心汤联合温针灸对心力衰竭患者中医证候积分及心功能的影响[J].辽宁中医杂志,2022,49(12):172-175. | Effects of Yiqi Jianxin Decoction Combined with Warm Acupuncture on Scores of TCM Syndromes and Cardiac Function in Patients with Heart Failure | Intervention was ineligible |
| 秦霞,朱帅兵,常文艳.温针灸背俞穴联合耳穴埋籽对老年心力衰竭患者心功能及生活质量的影响[J].实用中医内科杂志,2024,38(04):137-139. | Effect of Warming Acupuncture and Moxibustion at Back-Shu Acuoints Combined with Auricular Seed Implantation on Heart Failure and Quality of life of Elderly Patients with Heart Failure | Intervention was ineligible |
| 黄迎春,张明霞,贾金,等.针灸联合益心舒胶囊治疗老年慢性心力衰竭的临床研究[J].中西医结合心脑血管病杂志, 2024,22(11):2004-2007. | Clinical Study on Acupuncture Combined with Yixinshu Capsule in the Treatment of Elderly Patients with Chronic Heart Failure | Intervention was ineligible |
| 陈嘉文,王婷,周嘉宝,等.温针灸联合穴位敷贴对慢性心力衰竭患者最大通气量及血清髓过氧化物酶水平的影响[J].陕西中医,2025,46(06):844-847. | Effect of Warm Needling Combined with Acupoint Application Therapy on Maximal Voluntary Ventilation and Serum Myeloperoxidase Levels in Patients with Chronic Heart Failure | Intervention was ineligible |
| 谢悦悦,陈光瑞,张天宇,等.自拟益气活血方联合针灸治疗冠心病慢性心力衰竭临床观察[J].中医药临床杂志,2024,36(10):1948-1953. | Clinical Observation on the Treatment of Heart Function and Inflammatory Factors in Chronic Heart Failure of Coronary Heart Disease with Self made Yiqi Wenyang Recipe and Acupuncture and Moxibustion | Intervention was ineligible |
| 胡慧敏.体外反搏联合针灸对老年慢性心衰患者心功能的影响[J].中国疗养医学, 2021,30(03):288-290. | Effect of Enhanced External Counterpulsation Combined with Acupuncture on Cardiac Function in Elderly Patients with Chronic HF | Intervention was ineligible |
| 赵韶辉,杨立,王坤.益气复脉针联合腺苷注射液治疗心衰的临床疗效及对心功能和相关指标的影响[J].航空航天医学杂志,2023,34(01):68-71. | Clinical Efficacy of Yiqi Fumai Acupuncture Combined with Adenosine Injection in the Treatment of Heart Failure and Its Impact on Cardiac Function and Related Indicators | Intervention was ineligible |
| 王栋.益气温阳利水方联合针刺心俞穴对慢性心衰患者血清IL-17TNF-α的影响[J].四川中医,2015,33(12):47-49. | Effects of Yiqi Wenyang Lishui Formula Combined with Acupuncture at Xinshu (BL15) on Serum IL-17 and TNF-α Levels in Patients with Chronic Heart Failure | Intervention was ineligible |
| 郑晓峰,黄清玉,苏咸晶.无创正压通气联合针刺疗法对急性左心衰患者血气分析、NT-proBNP的影响[J].广西中医药, 2016,39(06):31-33. | Effects of Non-Invasive Positive Pressure Ventilation Combined with Acupuncture Therapy on Blood Gas Analysis and NT-proBNP in Patients with Acute Left Heart Failure | Intervention was ineligible |
| 高伟.养心活血针法联合西药治疗冠心病致慢性心衰临床研究[J].上海针灸杂志, 2017,36(06):676-678. | Clinical Study on Yang Xin Huo Xue Needling Method plus Western Medication for Chronic Heart Failure Due to Coronary Heart Disease | Intervention was ineligible |
| 程蕾群,许三雄.穴位二联疗法对慢性心力衰竭患者心功能及炎性因子的影响[J].针灸临床杂志,2017,33(12):16-19. | Effect of Acupoint Combination Therapy on Cardiac Function and Inflammatory Factors in CHF Patients | Intervention was ineligible |
| 黄铿儒,黄积存,陈业雄.丹参川芎嗪注射液联合“攻补”针刺疗法治疗冠心病合并慢性心力衰竭的临床观察[J].中国中医急症,2019,28(01):114-116. | Clinical Observation of Danshen Chuanxiongqin Injection Combined with the "Tonifying-Draining" Acupuncture Technique in the Treatment of Coronary Heart Disease Complicated with CHF | Intervention was ineligible |
| 刘亚红,赵越娟.生脉养心汤配合内关穴针刺对慢性心力衰竭患者心功能、炎性因子的影响[J].中医药信息, 2020,37(01):88-92. | Effect of Shengmai Yangxin Decoction Combined with Needling PC6 on Cardiac Function and Inflammatory Factors in Patients with CHF | Intervention was ineligible |
| 刘亚丽.温阳益心汤结合针刺对慢性心衰患者血清炎症因子水平的影响[J].光明中医,2020,35(12):1864-1866. | Effect of Wenyang Yixin Decoction Combined with Acupuncture on the Level of Serum Inflammatory Factors in Patients with Chronic Heart Failure | Intervention was ineligible |
| 叶敏谊,江慧仪,潘艳东.俞募配穴针刺联合西药治疗慢性心力衰竭的效果及对心衰标志物、心率变异性的影响[J].中医临床研究,2020,12(18):28-31. | Effect of acupuncture at back-shu and front-mu acupoints combined with western medicine in the treatment of chronic heart failure and its effect on heart failure markers and heart rate variability | Intervention was ineligible |
| 钟言,梁蕴瑜,卓剑丰,等.子午流注针法与八段锦联用对老年慢性心衰患者心脏康复的疗效分析[J].辽宁中医杂志,1-10[2026-01-03]. | Efficacy Analysis of the combination of Midday-Midnight Meridian-Flow Acupuncture and Baduanjin on Cardiac Rehabilitation in Elderly Patients with Chronic Herat Failure | research typewas ineligible |
| 师晓娜,刘云.针刺疗法联合多元化康复护理在CCU严重心力衰竭患者中的应用效果[J].实用中医内科杂志,2023,37(08):157-159. | The Application Effect of Acupuncture Therapy combined with DⅣersified Rehabilitation Nursing in Severe Heart Failure Patients in CCU | Intervention was ineligible |

**Supplementary Table S4.** Meta‑regression analyses

| indicators | Covariate | Univariate analysis | | | |  | Multivariate analysis | | |
| --- | --- | --- | --- | --- | --- | --- | --- | --- | --- |
|  |  | exp(β) | 95% CI | *P* | τ² |  | exp(β) | 95% CI | *P* |
| LVEF | Method | 2.1496 | -2.3045～6.6038 | 0.3442 | 7.369 | | 3.7385 | 1.3055～6.1714 | 0.0026 |
|  | Duration | 3.0336 | -0.9926～7.0599 | 0.1397 | 5.8199 | | 3.6605 | 1.3011～6.0198 | 0.0024 |
|  | Number | -0.8758 | -5.5991～3.8475 | 0.7163 | 8.5477 | | -1.43 | -3.89～1.0301 | 0.2546 |
|  | NYHA | -2.6239 | -7.3074～2.0595 | 0.2722 | 6.9098 | | -3.3318 | -5.6879～-0.9757 | 0.0056 |
| 6MWD | Method | -7.783 | -44.7121～29.1461 | 0.6796 | 420.91 | | 5.1665 | -51.0745 61.4074 | 0.8571 |
|  | Duration | 23.6272 | -4.6853～51.9396 | 0.1019 | 232.8307 | | 17.2835 | -38.9574 73.5245 | 0.547 |
|  | Number | -13.594 | -49.2554～22.0674 | 0.455 | 392.3727 | | -3.6335 | -59.8745 52.6074 | 0.8992 |
|  | NYHA | -35.0904 | -55.6717～-14.5092 | 0.0008 | 71.7228 | | -28.8665 | -109.1052 51.3723 | 0.4807 |

Abbreviations: LVEF: left ventricular ejection fraction; 6MWD: 6-minute walk distance; exp(β): exponentiated coefficient; τ²: heterogeneity variance.

**Supplementary Table S5.** LFK details and interpretation for each outcome

| Outcome | K | Lower random | Upper random | I^2^ | LFK index | judge |
| --- | --- | --- | --- | --- | --- | --- |
| LVEF | 7 | 2.274 | 7.678 | 0.847 | 2.156 | major asymmetry |
| 6MWD | 6 | 55.082 | 99.186 | 0.940 | 2.267 | major asymmetry |
| MLHFQ | 5 | -8.572 | -4.247 | 0.269 | -2.681 | major asymmetry |
| LVESD | 3 | -10.443 | -0.078 | 0.885 | 0.833 | no asymmetry |
| LVEDD | 4 | -7.790 | -2.043 | 0.689 | -0.851 | no asymmetry |

**Supplementary Appendix S1 GRADE evidence evaluation criteria**

**Factors that may reduce the level of quality of evidence:**

Each following question had 3 answers: “No serious risk”, “Serious risk” and “Very serious risk”.

Risk of Bias: “Serious risk” when about half of the studies had the lack of randomized methods or blinding.“Very serious risk” when almost all the studies had the lack of randomized methods, allocation concealment methods, and blinded evaluation.“No serious risk” when a few or none of the studies had the lack of randomized methods, allocation concealment methods, and blinded evaluation. Low-quality articles had a weak influence on the weight of the total combined effect

Inconsistency: “No serious risk” when I^2^≤50%; “Serious risk” when 50<I^2^≤75%; “Very serious risk” when I^2^>75%

Indirectness: According to PICO principle and Comprehensive consideration in combination with information.

Imprecision: “No serious risk” when the confidence intervals for the effect-size estimates were sufficiently narrow. “Very serious risk” when the confidence intervals for the effect-size estimates were wide. “Serious risk” when the widths of the confidence intervals for the effect-size estimates were in between.

Publication bias: “No serious risk” when absolute LFK index ≤ 1; “Serious risk”when 1 < absolute LFK index ≤ 2; “Very serious risk” when absolute LFK index > 2.

**Factors that may increase the level of quality of evidence:**

Plausible Confounding had 2 answers: “No” and “Yes”. It would be assessed by comprehensive consideration in combination with information.

Magnitude of effect had 3 answers: “No”, “Yes” and “Extremely” related to odds ratio (OR). “No” when 0.5<OR<2; “Yes” when 0.2<OR≤0.5 or 2≤OR<5; “Extremely” when OR≤0.2 or OR≥5.

There were 4 levels of quality: “High”, “Moderate”, “Low” and “Very low”. Evidence of RCTs were initially assessed as “High”. “Serious risk” would reduce 1 level of quality and “Very serious risk” would reduce 2 levels of quality. While “Yes” could promote 1 level of quality and “Extremely” could promote 2 level of quality.
